# Scalable Biosynthesis and Recovery of Poly-3-Hydroxybutyrate Produced from Cotton-Derived Glucose by *Cupriavidus necator*

**DOI:** 10.3390/polym17202745

**Published:** 2025-10-14

**Authors:** Ashley M. Clark, Lucia E. Gargano, Gabriella M. Fioravanti, Hannah M. Schapiro, Ronald G. Kander

**Affiliations:** School of Design & Engineering, Kanbar College, Thomas Jefferson University—East Falls, Philadelphia, PA 19063, USA; ashley.clark@students.jefferson.edu (A.M.C.); lucia.gargano@students.jefferson.edu (L.E.G.); gabriella.fioravanti@jefferson.edu (G.M.F.); hannah.schapiro@jefferson.edu (H.M.S.)

**Keywords:** biopolymer, bioreactor, P3HB, thermal analysis

## Abstract

To combat the growing issue of petroleum plastic waste, alternative bio-based polymers are being developed. Many of these biopolymers are made from bio-derived materials, or are biodegradable, but the most promising polymers fall in both categories. Polyhydroxyalkanoates (PHAs) are one such class of polymers, and poly-3-hydroxybutyrate (P3HB), the most popular PHA, has shown great potential. This study utilized two types of cotton-derived glucose, alongside commercial glucose, as a feedstock for the biosynthesis of P3HB by *Cupriavidus necator* (also known as *Ralstonia eutropha*). The fermentation took place in a 2-L bioreactor, showing potential for scale-up. A single-solvent extraction method was created and utilized to reduce process complexity and chemical consumption of the polymer extraction. Both cotton-derived glucoses were shown to produce more P3HB than commercial glucose. The resulting P3HB samples were compared to each other and to the literature based on polymer yield and thermal characteristics. While all samples averaged a smaller yield than seen in the literature (indicating the need for optimization of the bacterial growth and metabolism with a growth curve in our future work), the cotton-derived glucose was shown to yield more P3HB than commercial glucose. Further, cotton-derived P3HB had very similar thermal properties to the commercial glucose-derived P3HB (and to values from the literature) with onset of thermal degradation ranging from 185 °C to 263 °C, cold crystallization temperatures ranging from 24 °C to 28 °C, and melting temperatures ranging from 147 °C to 151 °C. Lastly, all samples were shown to have a similar percentage crystallinity, ranging from 38% to 45%, which is slightly lower than that reported in the literature. P3HB made from cotton-derived glucose was shown to have potential as a scalable, sustainable alternative process.

## 1. Introduction

Petroleum plastics litter the environment because of their improper use. Many products today utilize these materials because of their low cost and availability, but not for their long lifespans [1]. In 2018, the U.S. collected over 35 million tons of plastic municipal solid waste [2]; clearly, plastics are not being properly recycled or reused. There are some instances, as in the medical field during the COVID-19 pandemic, where long lasting, stable plastics are required for sterility and safety of single use products, but there is no reason such materials must be everywhere [3]. Making short-term or single-use products from petroleum plastics that consume precious petroleum and take thousands of years to degrade only creates litter, pollution, and excessive waste [1,4,5,6]. Even more, petroleum plastics in outdoor applications create microplastics as UV and weather begin to degrade them [4]. Then, these microplastics contaminate the air, soil, and water, and later, living organisms [7]. As a result, material developers have been working to create bioplastics as alternatives to mitigate the issue of petroleum plastic waste.

Bioplastics are polymers that are bio-based using bio-derived raw materials or are bio-degradable or compostable in a timely manner into non-harmful materials. Bioplastics of the most interest fall into both categories, some even utilizing biowaste as a raw material [8]. One high-value group of bioplastics, polyhydroxyalkanoates (PHAs), are being developed from waste biomass through microbial fermentation [4,8,9,10,11,12,13,14]. For example, from 2010–12 the European Commissions “ANIMPOL” project converted 500,000 tons per year of slaughterhouse waste (animal lipids) into 35,000 tons per year of PHA [9]. Instead of burning waste, valuable polymer was made.

The benefit of using waste biomass as opposed to raw biomass is the reduction of the final polymer cost. Raw material cost accounts for over 40% of total PHA production costs, making it the primary limiting factor of large-scale PHA production [8]. In 2023, the PHA market was expected to grow from $95 million to $195 million by 2028 [15]. For economically sustainable growth, every opportunity to reduce production costs must be taken so that PHAs may compete with the price-point of petroleum polymers.

As such, additional waste carbon sources have been explored. PHAs can be created from solid waste such as animal lipids, palm and olive oil effluent, and solids and wastewater from other agricultural products [9]. Plant-based agricultural waste can be used after it is transformed into glucose, as is done with hemp hurd [10]. Agricultural waste, given its constant production, appears to be a good carbon source for PHA, and potentially more economical sources of glucose exist besides hemp. Further cost reduction can occur by taking advantage of local biomass to limit transportation costs [8].

In the one-year period of August 2018 to July 2019, it is estimated that 1.3 million tons of crude fiber waste from carding, 7.8 million tons of cotton fiber waste from yarn production, and 3.8 million tons of cotton fabric waste from fabric production were generated by the global cotton and garment supply chain [16]. Combined, that is 12.9 million tons of global pre-garment cotton waste to create a final 14.6 million tons of cotton garments from 76.3 million tons of grown cotton globally [16]. According to the 2024 Monthly Economic Letter released by Cotton Incorporated, the leaders of cotton research and marketing in America, it is estimated that the U.S. produced 14.3 million 480-pound bales of cotton out of the 116.2 million produced worldwide in 2024 [17]; this puts the U.S. at 12.3% of global annual cotton grown and harvested in this past crop year. While not reported in the same year span, this percentage could be applied to the approximation of total cotton grown.

If 12.3% of 76.3 million tons of cotton grown annually world-wide was produced in the U.S., then 1.59 million tons of pre-garment cotton waste is created in the U.S. each year. That leaves a great supply of waste biomass to produce biopolymer. Enzymatic hydrolysis has been used to create glucose from pre-consumer cotton waste [16,18]. This glucose could be used to produce the most popular PHA, poly-3-hydroxybutyrate (P3HB).

P3HB is often produced from the fermentation of sugars using bacteria in growing conditions lacking specific nutrients and with a surplus of carbon [14]. This specific environment stresses the bacteria and causes them to store P3HB intracellularly as energy stores, much like animals store fat [13]. *Cupriavidus necator*, a common soil bacterium (also known by the name *Ralstonia eutropha*), is one of the best explored PHA producers [8]. Moreover, *C. necator*, also known by *Cupriavidus necator*, has been found to produce P3HB using glucose as the carbon feedstock [10].

Once a cost-effective carbon feedstock for P3HB production is chosen, other factors impacting the expense of production can be addressed. The complexity of the production process, including growth medium, growing conditions, and the demand for sterility, increases the manufacturing cost of PHA [12]. To reduce this cost, the scalability of P3HB production can be modeled after current practices in the pharmaceutical industry that utilize large-scale bioreactors for bacterial growth [19]. These established, sterile systems can be modified to fit P3HB production, as opposed to creating entirely new facilities or machines. This idea has been explored using small-scale, 2 L bioreactors and models of large-scale production [10,12]. Nonetheless, the conditions must be optimized given the specific carbon source, as the mechanisms used to convert the carbon into P3HB will vary [20].

The cost of current downstream polymer extraction methods also limits the industrial adoption of PHA production [8]. Polymer extraction requires the lysing of the cells to recover the intracellular biopolymer. Previous work has presented numerous methods for P3HB extraction [20]. While chloroform extraction has been explored extensively, it is typically used along with other chemicals [10,21]. This project uses a single-solvent extraction method with chloroform, where polymer is precipitated from chloroform via a rotary evaporator. Rotary evaporation, a well-known and scalable method, allows the chloroform to be collected and reused. To the authors’ knowledge, chloroform as a single solvent has yet to be applied in this setting.

A manufacturing process using a waste feedstock, an efficient bioreactor system, and recycled lysing solvent could produce P3HB at an attractive price point for an eco-friendly plastic. P3HB is sometimes preferred over the most widely used bioplastic, poly-lactic acid (PLA). P3HB is bio-based, compostable, and biodegradable in soil, water, and commercial environments, whereas PLA only degrades in commercial composting [9]. Further, because of its strength, hydrophobicity, inertness, relatively high melting point, optical purity, and processability, P3HB could replace many single-use plastic products [10]. Particularly, P3HB could replace polypropylene in many instances, as they share similar performance properties [14]. Also, polypropylene, as a petroleum-based thermoplastic, requires a chemically intensive synthesis. P3HB uses bacteria, and *C. necator* is a nontoxic, common soil bacterium, making the production process safer than that of petroleum plastics [10]. In use, P3HB shows promise as medical implants (sutures, scaffolds) and disposable food packaging [22].

This study aims to apply the three cost-reduction techniques described above to the production of P3HB with *C. necator*, using cotton-derived glucose as the carbon feedstock. P3HB production using two versions of pre-consumer cotton waste derived glucose solutions provided by Cotton Incorporated will be compared to production using commercial glucose. The fermentation will be carried out in a scalable, 2-L cylindrical stirred tank reactor (STR) and lysed using chloroform and a roto-evaporation technique designed to capture and recycle the chloroform. The final P3HB from the different glucose sources will be compared based on polymer yield, as well as on thermal properties.

## 2. Materials and Methods

### 2.1. Bacteria Preparation

*C. necator*, also known as *Cupriavidus necator*, was obtained from the American Type Culture Collection (ATCC) (ATCC17699, Manassas, VA, USA). The lyophilized cell pellet from ATCC was resuspended following the ATCC published handling procedure. The liquid culture was incubated for 48 h at 26 °C and 200 RPM. Then, the liquid culture was used for glycerol stocks. To make the glycerol stock, a 1:1 ratio of liquid culture and glycerol were resuspended and stored at −80 °C. A sample from the glycerol stock was sent to GENEWIZ (Azenta Life Sciences, Burlington, MA, USA) for 16S rRNA gene sequencing [23].

Agar plates were poured from a solution consisting of 0.75 g beef extract, 1.25 g peptone, 3.75 g of agar, and di H_2_O to make a total volume of 250 mL [14]. The agar plates were inoculated with 2–4 drops of the previously prepared liquid culture prior to its incubation, and were incubated for 48 h at 26 °C. After incubation, agar plates were stored at 4 °C and used to inoculate starter cultures for the bioreactor runs.

### 2.2. Media Preparation

A variety of different growth mediums were prepared and used during the growing procedure, including nutrient broth, trace salt solution, mineral salt medium (MSM) kept as two parts (MSM1 and MSM2), commercial glucose (G), control cotton-derived glucose solution (CCG), and cutting waste cotton-derived glucose solution (CWG).

The nutrient broth was made with 0.75 g beef extract, 1.25 g peptone, and finished with di H_2_O to make a total volume of 250 mL [10].

The trace salt solution was made in a 100× concentration with 100 mg/L ZnSO_4_-7H_2_O, 30 mg/L MnCl_2_-4H_2_O, 300 mg/L H_3_BO_3_, 200 mg/L CoCl_2_-6H_2_O, 10 mg/L CuCl_2_-6H_2_O, 20 mg/L NiCl_2_-6H_2_O, and 30 mg/L NaMoO_4_-2H_2_O and finished with diH_2_O for a total volume of 100 mL [14].

The mineral salt medium (MSM) was made in a 5× concentration in two parts to prevent phosphate precipitation. Part one (MSM1) consisted of 1.25 g NH_4_Cl, 0.25 g MgSO_4_, and 3 mg (NH_4_) Fe(SO_4_)_2_–12H_2_O, for a total volume of 250 mL [14]. Part two (MSM2) consisted of 7.5 g Na_2_HPO_4_ and 1.25 g KH_2_PO_4_, for a total volume of 250 mL [14]. The two MSM formulations were maintained at a neutral pH using NaOH (1 M). All solutions, along with 1 L of di H_2_O, were autoclaved at 121 °C for 30 min. Once cool, MSM1 and MSM2 both received 0.125 mL of the trace salt solution, making each solution 0.05% *v*/*v* trace salts [14].

The commercial glucose solution (G) was prepared according to Khattab [10]. In short, 80 g granulated D-glucose (Thermo Fischer Scientific, Waltham, MA, USA) was dissolved in 1 L di H_2_O, and the resultant solution was sterile filtered through a 0.2 µm PTFE membrane. The cotton-derived glucose solutions were received from Cotton Incorporated. Control cotton glucose solution (CCG) was produced from Cotton Incorporated’s own preproduction cotton waste, while cutting waste cotton glucose solution (CWG) was made from a major textile brand’s cotton fabric scrap. All solutions were made by Cotton Incorporated using enzymatic hydrolysis and were heated to 130 °C after hydrolysis to denature the enzyme [18]. Upon receiving the solution, a sterile filter with a 0.2 µm PTFE membrane was used to remove contaminants and remaining enzymes. The sterile solutions were kept at 4 °C to reduce the risk of microbial contamination. The weight percent concentration of glucose for each solution was determined to find the correct dilutions necessary for the growth media. All glucose solutions were tested by Cotton Incorporated to measure glucose concentration and verified in our laboratory upon receipt of each batch. The solutions contained only glucose and showed no indication of other sugars or trace minerals per Cotton Incorporated’s reported testing. This was confirmed with spot NMR testing in our laboratory. Starting glucose solution concentrations as used in this study are reported in Table 1 and Table 2.

### 2.3. Starter Culture Preparation

All procedures were completed within a sterile field. Starter cultures, containing a total of 5 mL of a 50/50 solution of nutrient broth and MSMs with various glucose sources, were prepared as shown in Table 1. Each inoculum tube then received a swipe of the *C. necator* bacterial lawn from the previously prepared agar plates and was incubated in a shaking incubator at 30 °C and 200 RPM for 72 h. This pre-fermentation inoculum growth was essential to acclimate the bacteria to using glucose as the carbon source for metabolism; it has been found that *C. necator* takes approximately 72 h to get through the lag phase of growth while using glucose [10]. The inoculum media was 50% nutrient broth, as shown in Table 1, to encourage growth and establishment as the bacteria adjusted to the glucose carbon source. While *C. necator* H16 can only use fructose as a feedstock and cannot assimilate glucose, a glucose-positive phenotype can be isolated after 70 h when grown on media with high glucose concentration [11].

### 2.4. Bioreactor Procedure

A 2-L stirred tank bioreactor (Sartorius Stedim Biostat A, Göttingen, Germany) was used for cell fermentation. All parts of the reactor were autoclaved at 121 °C for 30 min in preparation for use. The set-up was carried out according to an SOP designed and verified by Jefferson’s Institute of Bioprocessing [24]. The starter culture, as described above, was used to inoculate the bioreactor according to Table 2. Roughly 15 mL of Antifoam B Emulsion, aqueous s-silicone emulsion (Sigma Aldrich, St. Louis, MO, USA), was deposited into the solution prior to the start of fermentation to prevent the foaming caused by agitation in the bioreactor. Batch fermentation was carried out for 72 h, at 30 °C, with agitation at 400 RPM and an airflow at 300 ccm. These conditions were selected to operate at conditions near those used in the literature and imitate what might be experienced in shake flask experiments [14] (greater agitation speeds may be explored in future studies to optimize biomass and polymer yields). The air used to provide the system with oxygen was breathing grade air (air–gas), which has 76.5–80.5% nitrogen and 19.5–23.5% oxygen. All bioreactor runs were performed with a glucose concentration of 80 g/L (after isolating the glucose-positive phenotype during the starter culture preparation described above). This glucose concentration led to good biomass growth and allowed for reasonable polymer yields in this proof-of-concept study. Further optimization of the glucose concentration will be included in our future work.

### 2.5. Polymer Extraction

After 72 h, the solutions were collected and centrifuged at 3000 RPM and 5 °C for 10 min. The supernatant was poured off and the cell pellets of identical growing conditions were combined into a 500 mL round bottom, multi-neck glass vessel. This glass vessel was placed in an oven at 50 °C to completely dry for 48–96 h. This dry weight was recorded.

The volume of the required amount of chloroform was chosen based on Khattab’s methods as a 1:4 *w*/*v* ratio [10]. Under a fume hood, the chloroform was measured and poured into the glass vessel with the cell pellet. If the pellet appeared stuck to the bottom of the vessel, it was scraped up to expose as much of the cells as possible to the chloroform. The solution was ultrasonicated for 10 min on low power. After sonication, the flask was placed on a heating mantle that was set to 62 °C to boil the solution for two hours. Next, the chloroform-cell solution was poured through a ceramic filter and filtered via vacuum with coarse P8 creped filter paper into an Erlenmeyer flask. The filter paper was set aside to dry for weight of the collected cell biomass. If there were cell particles stuck in it, the round bottom flask was also left to dry and weighed. The filtered chloroform solution was then poured into a single neck round bottom and placed on a Rotary Evaporator with a water bath set to 40 °C until all the chloroform evaporated, and the polymer was left as a film on the walls of the flask.

### 2.6. Solvent Cast Film Production

The biopolymer was peeled from the walls of the round bottom and collected into a small flat bottom glass evaporation dish, 7 cm in diameter. Polymer samples weighing between 0.2 and 0.3 g were dissolved in 40–50 mL of chloroform until the polymer redissolved. The dish was then placed on a hot plate set to 100 °C, and a slightly larger flat bottom evaporation dish was placed over the top to create an oven-like environment with dispersed heat. The solution was allowed to boil until most of the chloroform had evaporated. The temperature was then reduced to 50 °C and left to completely dry. Once all the chloroform evaporated off, the solvent cast film was removed and stored in an airtight container. Polymer film thicknesses ranged from 0.06 and 0.15 mm, as measured with precision calipers.

### 2.7. Thermal Analysis

Thermogravimetric analysis (TGA) (TA Instruments Q50 TGA, New Castle, DE, USA) and differential scanning calorimetry (DSC) (TA Instruments Q200 DSC) tests were performed on the produced polymer, as well as synthetic P3HB purchased from Sigma-Aldrich, to confirm the material made and assess future processing parameters. Specimens weighing approximately 5 mg were cut from solvent-casted films and used for all thermal analysis testing. All TGA ramp tests were run from 0 °C to 350 °C at 10 °C/min with a 30 mL/min nitrogen gas flow rate. All DSC heat-cool-heat tests were run from −20 °C to 200 °C at 10 °C/min with a 30 mL/min nitrogen gas flow rate.

Onset and end temperatures, peak temperatures, and enthalpy values were derived from the resulting graphs using TRIOS software (V. 5.2) (TA Instruments Waters). Reported values were rounded to the nearest whole number for ease of understanding, while calculations were conducted using exact values. Crystallization percentage was calculated according to De Sousa Junior, using the following formula:

$$X\left(\%\right)\;=\;\frac{∆H}{∆H_{m}^{0}}\times \;100,$$

where Δ*H* is the melting or crystallization enthalpy of the sample, and $∆H_{m}^{0}\;=\;146$ J/g, the melting enthalpy of 100% crystalline P3HB [25].

## 3. Results and Discussion

### 3.1. Confirmation of C. necator in Culture and Film Casting

**Figure 1:**
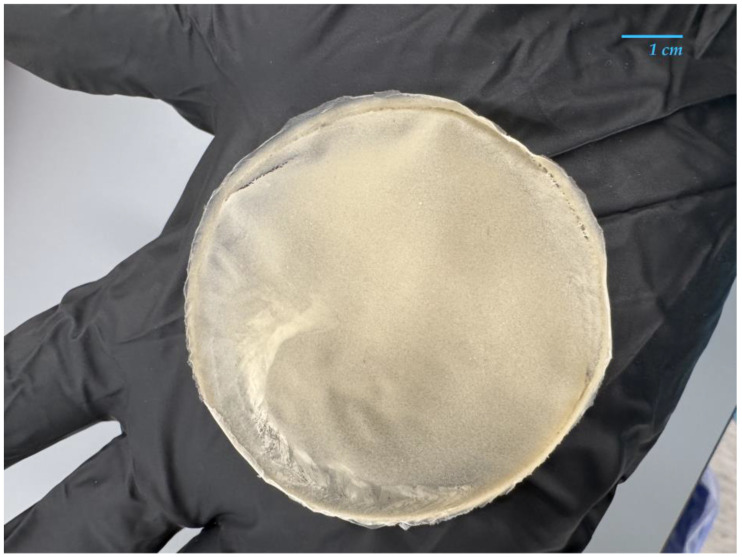
One P3HB film created using chloroform-only extraction and solvent casting method.

To confirm sterile growth conditions and the presence of *C. necator*, samples from the starter culture were sent out for analysis (Azenta Life Sciences, Burlington, MA, USA). Gene sequencing of 16S rRNA confirmed that *C. necator* was the only species present in the sample. After fermentation of *C. necator* and polymer extraction using the single-solvent method, P3HB was successfully recovered from both G-fueled growth and cotton glucose-fueled growth. This finding was confirmed with thermal analysis and is the first time P3HB in a bioreactor has been grown with this carbon source. The chloroform-only extraction method separated the P3HB from the *C. necator*, showing the polymer could be recovered with a solvent that is easily collected and reused. Figure 1 is an example of one of the P3HB films cast directly from the chloroform solution. Cast films will be used to measure mechanical properties as part of our ongoing work.

### 3.2. P3HB Yields

The literature presents a vast array of yield percentages of P3HB from *C. necator.* Khattab, using a growth method much like this study, but an alternate glucose source and extraction process, found a P3HB yield of 56.5% [10]. Kovalick noted yields from many different carbon sources, including sugars and oils ranging from 46.3% to 77.1% [12].

In comparison to the literature, the P3HB yields from this study are much lower, but do show some value within. As displayed in Table 3, the polymer yields from cotton-derived glucoses are much greater than those of the P3HB from commercial glucose. The CCG allowed the bacteria to create the most P3HB, with an average yield of 27.8 ± 15.2%, and from CWG there was an average yield of 16.6 ± 10.2%. Given the commercial glucose created only an average polymer yield of 1.30 ± 0.86%, it is reasonable to assume that the glucose source had an effect. The maximum G-derived P3HB yield of 2.62%, does not even fall within the standard deviation of either cotton-derived P3HB yields, thus the glucose source likely affected how the *C. necator* were able to produce the P3HB. It is interesting to note though that the cotton-derived polymer yields gave a much higher standard deviation, thus a much larger range; more replications with the cotton-derived glucose and deeper statistical analysis would help determine why this is so.

### 3.3. Thermal Analysis

The initial goal of thermal analysis was to confirm the production of P3HB, as opposed to another PHA polymer. While the *C. necator*, growth environment, and glucose carbon source have all been shown by Khattab and others to produce specifically P3HB, it was imperative to confirm this result, especially given the use of an alternate glucose source [10]. To confirm the production of P3HB, polymer derived from all three glucose sources underwent thermal analysis via thermogravimetric analysis (TGA) and differential scanning calorimetry (DSC). The thermal metrics used to confirm the production of P3HB include the temperature of the onset of degradation (T_onset_), given by TGA, and the melting temperature (T_m_), given by DSC.

**Figure 2:**
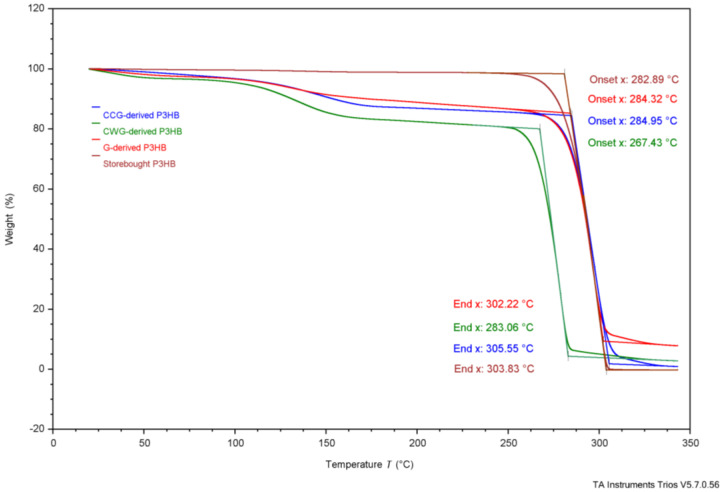
Thermogravimetric analysis results of P3HB produced using G, CCG, and CWG, and of the commercial, purchased P3HB.

As shown in Table 4, reported T_onset_ temperatures range from 185 °C to 263 °C. Figure 2, the TGA results, show that G-derived P3HB had a T_onset_ of 284 °C, CCG-derived P3HB had a T_onset_ of 285 °C, CWG-derived P3HB had a T_onset_ of 267 °C, and the purchased P3HB had a T_onset_ of 283 °C. The T_m_ values for P3HB found in the literature range from 170 to 180°; the T_m_ values measured were 147 °C for G-derived P3HB, 151 °C for CCG-derived P3HB, 150 °C for CWG-derived P3HB, and 165 °C for the purchased P3HB. The T_onset_ of the tested samples falls just above the range of the literature values, and the T_m_ values fall just below or within, suggesting they likely are all P3HB.

Factors in production, especially the method of lysing, can affect the resulting P3HB polymer morphology [26]. The slight differences in the degradation and melting temperatures can be attributed to the variance in polymer morphology.

Thermal analysis can also provide details for potential processing of the P3HB as well. Typically, the melting temperature (T_m_) and the temperature of the onset of degradation (T_onset_) of P3HB are in proximity, making processing at a precise temperature extremely important [27]. Further, P3HB typically degrades quickly, making it important to not begin degradation even slightly while processing P3HB [27].

**Table 4:** Collected thermal analysis data for each experimental P3HB sample compared to the literature values, from Figure 2, Figure 3, Figure 4, Figure 5 and Figure 6.

Test	Property	G-Derived P3HB	CCG- Derived P3HB	CWG- Derived P3HB	Purchased P3HB	Literature Source				
						Wang [14]	Mannina [5]	Lee [28]	Sudesh [29]	Rysbek [13]
TGA	T_onset_	284 °C	285 °C	267 °C	283 °C	263.4 °C	185 °C	200 °C	-	-
	T_end_	302 °C	306 °C	283 °C	304 °C	269.8 °C	267 °C	-	-	-
DSC	T_g_	-	-	-	-	-	-	5 °C	4 °C	-
	T_c_ *	37 °C	36 °C	26 °C	73 °C	-	-	-	50–60 °C	-
	T_cc_ *	26 °C	24 °C	28 °C	-	-	-	-	-	-
	T_m_ *	147 °C	151 °C	150 °C	165 °C	172.05 °C	-	175 °C	180 °C	170 °C

**Figure 3:**
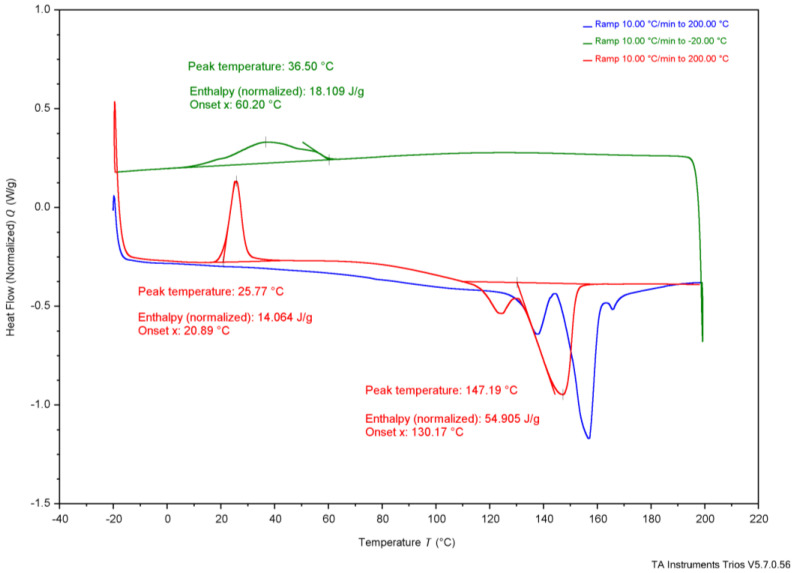
Differential scanning calorimetry results of P3HB produced using G.

**Figure 4:**
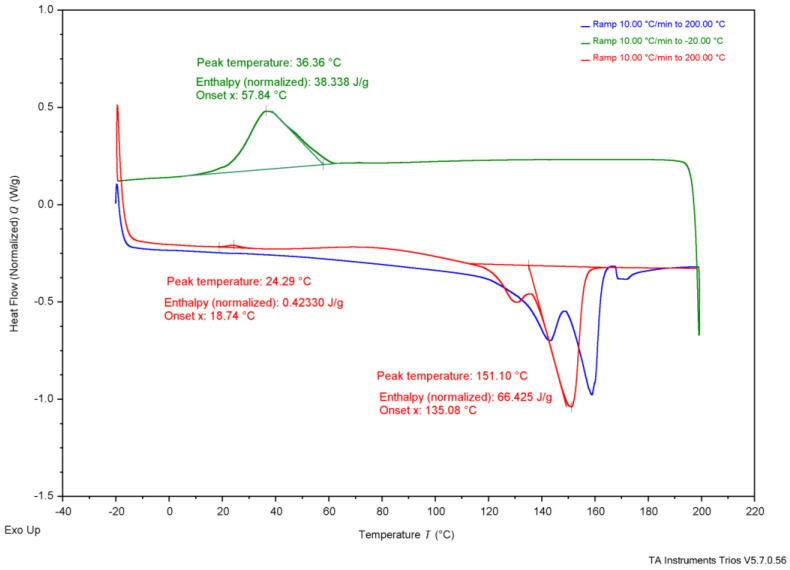
Differential scanning calorimetry results of P3HB produced using CCG.

**Figure 5:**
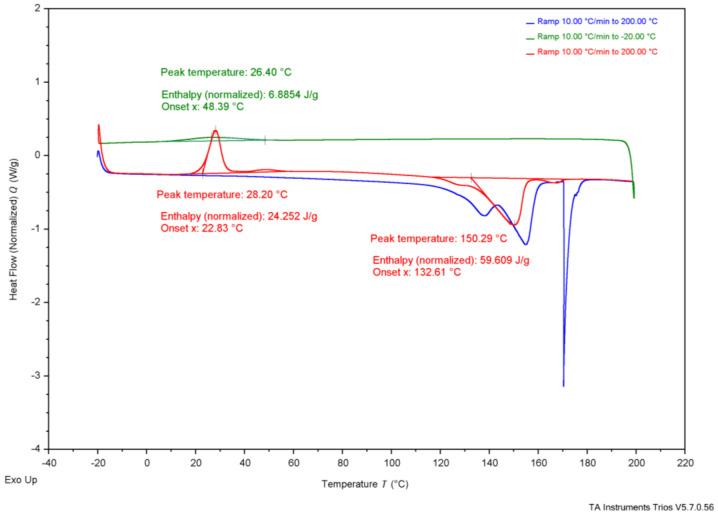
Differential scanning calorimetry results of P3HB produced using CWG.

**Figure 6:**
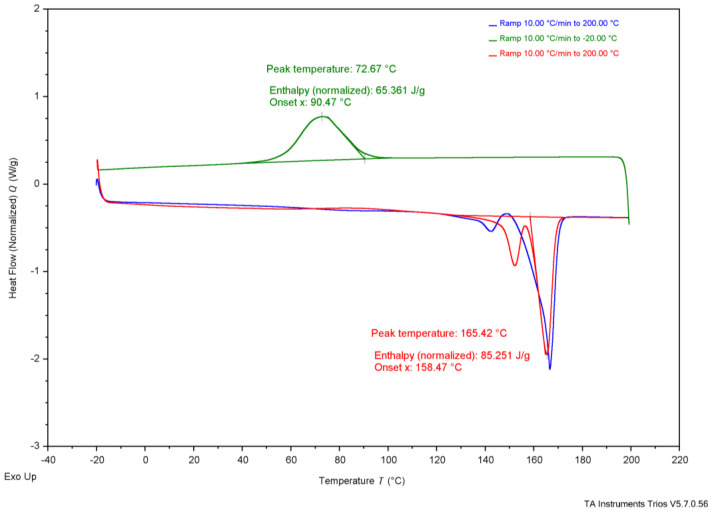
Differential scanning calorimetry results of commercial, purchased P3HB.

* T_c_ values are determined from the crystallization peak on the cooling curve, T_cc_ values are determined from the crystallization peak on the second heat curve, and Tm values are determined from the melting peak on the second heat curve; see Figure 3, Figure 4, Figure 5 and Figure 6 for the DSC curves.

Interestingly, as shown in Table 4, there is over 100 °C between the T_m_ and T_onset_ of the G-derived P3HB and both cotton sugar-derived P3HBs. The differences for G-derived P3HB and CCG-derived P3HB are nearly equal, with the difference for CWG-derived P3HB only about 15 °C smaller. Regardless of the glucose source, all produced P3HB samples show a larger gap between T_m_ and T_onset_ than seen in the literature (Table 4). This suggests that the bioprocessing and extraction methods used created a larger window available to select a temperature at which the polymer could flow and be processed via molding, compounding, extrusion, etc., without causing any degradation. Therefore, this production method might allow for more flexibility in temperature, bringing slight ease to processing by reducing the necessary control.

While the processing window was found to be different than that seen before, the previously reported rapid degradation after T_onset_ was observed, as shown in Figure 2. The CWG-derived P3HB showed the most rapid degradation, with G-derived P3HB then CCG-derived P3HB coming shortly behind. Thus, care must be taken to control the resonance time in manufacturing equipment when processing glucose-derived P3HB.

Further, thermal analysis is often used to explain how a polymer might behave in daily use. The glass-transition temperature (T_g_), typically shown on a DSC graph, represents the value at which the amorphous regions of the polymer switches from a more glassy, brittle state to a more rubbery, flexible state. If T_g_ is below the typical ambient temperature of 20 °C, the polymer is used regularly when its amorphous regions are rubbery, whereas if T_g_ is above ambient temperature, the amorphous regions of the polymer are glassy. T_g_ is not apparent in the DSC graphs from the produced P3HB, shown in Figure 3, Figure 4 and Figure 5, likely due to the T_c_ curve coming so close, but the literature establishes the T_g_ of P3HB at about 4–5 °C [28,29]. This places the amorphous regions of P3HB in a rubbery state at ambient temperature. P3HB is known to be a highly crystalline and brittle polymer [14]. The brittleness of the polymer is due to the high crystallinity, as the amorphous regions are existing in the rubbery state. Therefore, P3HB that is less crystalline, and thus more amorphous, should be less brittle than P3HB with higher crystallinity.

The P3HB produced using the methods described above are much less crystalline than the purchased P3HB and the P3HB reported on by Wang; see Table 5 [14]. The total melt polymer crystallinity, X_m_, derived from Wang’s reported ΔH_m_ value, comes to 61% crystalline [14]. The purchased P3HB was calculated to be 58% crystalline, while the G-derived P3HB and both cotton glucose-derived samples were found to be 38–45% crystalline. This suggests that the method used to create films of the produced polymer created a less crystalline, and likely less brittle P3HB, potentially reducing the common downfall of brittleness in use. Further investigation, including mechanical analysis, is needed to support this claim, but the idea shows potential.

The double melting peaks shown in all tested samples are also interesting to note. While all produced samples showed this behavior, it is not necessarily always shown in the literature. The DSC of P3HB presented by Wang shows a single sharp melting peak [14]. Some DSC curves by Janigová show double peaks, while others do not [27]. The two peaks indicate a secondary crystal structure within the P3HB samples, which must be a result of the production or extraction methods if said structure is not always seen. Further analysis of the crystal structures would be required to understand why such variances may be forming and whether they also influence the mechanical behavior of the P3HB samples.

### 3.4. Comparing Experimental Samples

While comparing the produced samples to the literature values makes them appear quite similar, there are many differences. Firstly, as seen in the TGA results, shown in Figure 2, the T_onset_ of CWG-derived P3HB comes about 17 °C before that of G-derived and CCG-derived P3HB, and the T_end_ comes about 20 °C before either sample. These data suggest that P3HB grown from the CWG is slightly less thermally stable due to the lower onset of degradation and end of degradation temperature. The lower T_onset_ and T_end_ could be caused by polymer morphology, like a slightly lower molecular weight or different crystal structure due to the alternate glucose source, or simply due to slight variances in the growth process and film casting. Further characterization is needed to support any such conclusions.

Secondly, the transitions seen on the DSC graphs, shown in Figure 3, Figure 4 and Figure 5, offer differences. The temperature of crystallization from the melt (T_c_), of G-derived P3HB and CCG-derived P3HB are about 10 °C higher than T_c_ of CWG-derived P3HB, Table 4. Further, the related enthalpy (ΔH_c_), and thus the percent crystallization from the melt (X_c_), of CWG-derived P3HB is much smaller than that of the other two samples, shown in Table 5. This suggests that CWG-derived P3HB is a slower crystallizer from the melt, as all three samples were cooled at the same rate during the DSC runs; see Figure 3, Figure 4 and Figure 5. Slower melt crystallization requires a polymer to be cooled slower during processing, meaning CWG-derived P3HB may take longer to form products with an equal amount of crystallinity as those made from the other two samples.

Because the CWG-derived polymer crystallized much less in the melt, the cold crystallization percent (X_cc_) was much higher than in the other two samples, shown in Table 5. Regardless, cold crystallization (T_cc_) of all three samples occurred at the same point, ranging only from 24 °C to 28 °C; see Table 4. At the end though, all three samples melted at nearly the same temperature, ranging from 147 °C to 151 °C (Table 4), and were quite similar in total crystallization percentage, from 38% to 45% (Table 5). Further, the X_c_ and X_cc_ of all three samples only explained about 50% of the total X_m_ (Table 5); this is interesting to note, as it suggests the method of calculating X_c_ and X_cc_ using the standard melting enthalpy of P3HB of 146 J/g is not ideal, and investigation into the effect of temperature on the standard enthalpy of P3HB could be explored. Regardless, given the final X_m_ values, all three materials should behave the same when made into a final product, though mechanical analysis would help confirm this idea.

## 4. Future Work

Future work includes a mechanical analysis of the P3HB produced using cotton-derived glucose. An AMDET micro tester along with MTEST Quattro software (Version 5.03.01) will be used to perform tensile testing on P3HB from each glucose source to compare the material to P3HB produced elsewhere, as well as to other polymers, to suggest potential end uses. It should be noted that yields in this proof-of-concept study were relatively low compared with the literature, especially for the commercial glucose runs. Therefore, a study on the optimization of polymer production in the bioreactor will be performed next by monitoring the bacterial growth and metabolism with a growth curve. The addition of an antibiotic into the growth media, and thus an antibiotic resistance gene into the bacteria, may also be explored, to reduce the possibilities of contamination once upscaled for more economical production.

## 5. Conclusions

To contribute to the goal of producing a more economically viable biopolymer, this study focused on making the bioderived and biodegradable poly-3 hydroxybutyrate (P3HB) through the fermentation of *C. necator* fueled by cotton-derived glucose in a bioreactor. A single-solvent extraction method was created and utilized to reduce the process complexity and chemical consumption of the polymer extraction. Both cotton-derived glucoses were shown to produce more P3HB than commercial glucose. CCG-derived polymer showed very similar thermal properties to G-derived P3HB, and CWG-derived P3HB was only slightly less thermally stable. Further, both cotton glucose-derived polymer samples had similar total crystallinity to G-derived P3HB, meaning they should perform similarly as final materials. That, along with the developed process resulting in P3HB with a larger processing temperature window and a lower total crystallinity than P3HB reported in the literature, suggests that this method, in combination with the use of cotton-derived glucose, makes a promising P3HB polymer. Further exploration on manufacturing and implementation into the world of existing polymers is warranted.

## Figures and Tables

**Table 1:** Preparation of 5 mL starter culture solutions.

Growth Medium	Control	G	CCG	CWG
Nutrient Broth	5 mL (100% *v*/*v*)	2.5 mL (50% *v*/*v*)	2.5 mL (50% *v*/*v*)	2.5 mL (50% *v*/*v*)
MSM 1 (5× concentration)	-	0.25 mL (5% *v*/*v*)	0.25 mL (5% *v*/*v*)	0.25 mL (5% *v*/*v*)
MSM 2 (5× concentration)	-	0.25 mL (5% *v*/*v*)	0.25 mL (5% *v*/*v*)	0.25 mL (5% *v*/*v*)
Commercial Glucose Solution (7.4% *v*/*w* glucose)	-	0.625 mL (12.5% *v*/*v*)	-	-
Cotton Glucose Solution (8.93% *v*/*w* glucose)	-	-	0.518 mL (10.36% *v*/*v*)	-
Cutting Waste Cotton Glucose Solution (9.06% *v*/*w* glucose)	-	-	-	0.510 mL (10.2% *v*/*v*)
di H_2_O	-	1.375 mL (27.5% *v*/*v*)	1.482 mL (29.64% *v*/*v*)	1.49 mL (29.8% *v*/*v*)

**Table 2:** Preparation of 1 L Bioreactor Fermentation Solutions.

Growth Medium	G	CCG	CWG
MSM 1 (5× concentration)	95 mL (9.5% *v*/*v*)	95 mL (9.5% *v*/*v*)	95 mL (9.5% *v*/*v*)
MSM 2 (5× concentration)	95 mL (9.5% *v*/*v*)	95 mL (9.5% *v*/*v*)	95 mL (9.5% *v*/*v*)
Commercial Glucose Solution (7.4% *v*/*w* glucose)	498.75 mL (49.875% *v*/*v*)	-	-
Cotton Glucose Solution (8.93% *v*/*w* glucose)	-	413.3 mL (41.33% *v*/*v*)	-
Cutting Waste Cotton Glucose Solution (9.06% *v*/*w* glucose)	-	-	407.368 mL (40.737% *v*/*v*)
di H_2_O	261.25 mL (26.125% *v*/*v*)	346.7 mL (34.67% *v*/*v*)	352.632 mL (35.263% *v*/*v*)
Starter Culture Inoculum	50 mL (5% *v*/*v*)	50 mL (5% *v*/*v*)	50 mL (5% *v*/*v*)

**Table 3:** P3HB yield data grown from respective sugar sources in the 2-L bioreactor.

Glucose Source	Run	Cell Dry Weight (CDW) (g) *	P3HB Yield (g)	P3HB Yield (%) **
Commercial Glucose (G)	G1	5.86 g	0.10 g	1.69%
	G2	5.05 g	0.03 g	0.59%
	G3	6.54 g	0.06 g	0.92%
	G4	3.81 g	0.10 g	2.62%
	G5	4.50 g	0.03 g	0.67%
	Mean ± Std. Dev.	-	-	1.30 ± 0.86%
Control Cotton Glucose (CCG)	CCG1	3.07 g	1.14 g	38.5%
	CCG2	6.4 g	1.09 g	17.0%
	Mean ± Std. Dev.	-	-	27.8 ± 15.2%
Cutting Waste Cotton Glucose (CWG)	CWG1	3.21 g	0.9 g	28.0%
	CWG2	6.05 g	0.82 g	13.6%
	CWG3	9.41 g	0.78 g	8.29%
	Mean ± Std. Dev.	-	-	16.6 ± 10.2%

* Dried cell matter with intracellular P3HB pre-lysing. ** P3HB Yield (%) = [P3HB Yield (g)/CDW (g)] × 100.

**Table 5:** Enthalpy and percent crystallization of prepared samples and purchased P3HB from Figure 3, Figure 4, Figure 5 and Figure 6.

Property	G-Derived P3HB	CCG- Derived P3HB	CWG- Derived P3HB	Purchased P3HB	Wang [14]
ΔH_c_ J/g	18 J/g	38 J/g	6.9 J/g	65 J/g	-
X_c_ (%)	12.3%	26.0%	4.7%	44.5%	-
ΔH_cc_ J/g	14 J/g	0.42 J/g	24 J/g	-	-
X_cc_ (%)	9.6%	0.3%	16.4%	-	-
ΔH_m_ J/g	55 J/g	66 J/g	60 J/g	85 J/g	89.7 J/g
X_m_ (%)	37.7%	45.2%	41.1%	58.2%	61.4%

## Data Availability

The original contributions presented in this study are included in the article. Further inquiries can be directed to the corresponding author.

## Abbreviations

The following abbreviations are used in this manuscript:

P3HB	Poly-3-hydroxybutyrate
G	Glucose, referring to commercial glucose
CCG	Cotton glucose
CWG	Cutting waste cotton glucose
